# Genomic expansion of magnetotactic bacteria reveals an early common origin of magnetotaxis with lineage-specific evolution

**DOI:** 10.1038/s41396-018-0098-9

**Published:** 2018-03-26

**Authors:** Wei Lin, Wensi Zhang, Xiang Zhao, Andrew P. Roberts, Greig A. Paterson, Dennis A. Bazylinski, Yongxin Pan

**Affiliations:** 1 Key Laboratory of Earth and Planetary Physics, Institute of Geology and Geophysics, Chinese Academy of Sciences, Beijing, 100029 China; 2 Institutions of Earth Science, Chinese Academy of Sciences, Beijing, 100029 China; 3 France-China Joint Laboratory for Evolution and Development of Magnetotactic Multicellular Organisms, Chinese Academy of Sciences, Beijing, 100029 China; 4 College of Earth Sciences, University of Chinese Academy of Sciences, Beijing, 100049 China; 5 Research School of Earth Sciences, Australian National University, Canberra, ACT 2601 Australia; 6 Department of Earth, Ocean and Ecological Sciences, University of Liverpool, Liverpool, L69 7ZE UK; 7 School of Life Sciences, University of Nevada at Las Vegas, Las Vegas, NV 89154-4004 USA

## Abstract

The origin and evolution of magnetoreception, which in diverse prokaryotes and protozoa is known as magnetotaxis and enables these microorganisms to detect Earth’s magnetic field for orientation and navigation, is not well understood in evolutionary biology. The only known prokaryotes capable of sensing the geomagnetic field are magnetotactic bacteria (MTB), motile microorganisms that biomineralize intracellular, membrane-bounded magnetic single-domain crystals of either magnetite (Fe_3_O_4_) or greigite (Fe_3_S_4_) called magnetosomes. Magnetosomes are responsible for magnetotaxis in MTB. Here we report the first large-scale metagenomic survey of MTB from both northern and southern hemispheres combined with 28 genomes from uncultivated MTB. These genomes expand greatly the coverage of MTB in the *Proteobacteria*, *Nitrospirae*, and *Omnitrophica* phyla, and provide the first genomic evidence of MTB belonging to the *Zetaproteobacteria* and “*Candidatus* Lambdaproteobacteria” classes. The gene content and organization of magnetosome gene clusters, which are physically grouped genes that encode proteins for magnetosome biosynthesis and organization, are more conserved within phylogenetically similar groups than between different taxonomic lineages. Moreover, the phylogenies of core magnetosome proteins form monophyletic clades. Together, these results suggest a common ancient origin of iron-based (Fe_3_O_4_ and Fe_3_S_4_) magnetotaxis in the domain *Bacteria* that underwent lineage-specific evolution, shedding new light on the origin and evolution of biomineralization and magnetotaxis, and expanding significantly the phylogenomic representation of MTB.

## Introduction

Earth’s global magnetic field provides a pervasive and valuable reference frame that diverse organisms use for both short- and long-distance navigation and migration. The origin of this navigational capability, known as magnetoreception [1] or, in prokaryotes and protozoa, magnetotaxis [2], is regarded as a major evolutionary innovation in the history of life. Magnetotactic bacteria (MTB) biomineralize intracellular, membrane-bounded, nano-sized magnetic mineral crystals of magnetite (Fe_3_O_4_) and/or greigite (Fe_3_S_4_) called magnetosomes and are characterized by their ability to sense and swim along Earth’s magnetic field lines [3]. Magnetosomes are the only magnetoreceptors definitively located at a specific site within cells so far and are a sufficiently well-characterized system with which the origin and evolution of magnetotaxis can be explored [4, 5].

It has previously been suggested that magnetotaxis, based on the biomineralization of Fe_3_O_4_ and Fe_3_S_4_ in magnetosomes, in different bacterial lineages evolved independently and that MTB originated polyphyletically [6]. However, this conclusion was made prior to the identification of conserved magnetosome gene clusters (MGCs) responsible for magnetosome biomineralization and magnetotaxis in both Fe_3_O_4_- and Fe_3_S_4_-producing MTB, which suggests that magnetotaxis in bacteria originated only once, so that it has a monophyletic origin [7–12]. Recent phylogenetic analyses suggest an ancient origin of bacterial magnetotaxis in the Archean Eon, thereby making this behavior a primal physiological process and possibly one of the first examples of biomineralization on early Earth [13]. However, due to the patchy distribution of MTB across different phylogenetic lineages, it has been difficult to infer the evolution of magnetotaxis in prokaryotes. Whether the genes for magnetotaxis have been transferred extensively horizontally between different microorganisms or been mainly inherited through vertical transfer remains unknown [14–16].

Although MTB have been known to exist for nearly half a century, current phylogenetic information on them is based primarily on 16S rRNA gene sequences, only a small fraction of which are represented by axenic cultures [15]. For a number of years, MTB were thought to phylogenetically only belong to the *Alphaproteobacteria*, *Deltaproteobacteria*, and *Gammaproteobacteria* classes of the *Proteobacteria* phylum and the *Nitrospirae* phylum [17–20]. Recent new evidence has revealed previously unknown, uncultured MTB to be affiliated with the candidate phylum *Omnitrophica* (previously known as candidate division OP3), the candidate phylum *Latescibacteria* (previously known as candidate division WS3), and the phylum *Planctomycetes*, thereby suggesting that magnetotaxis is likely more widespread in the domain *Bacteria* than previously thought [16, 21, 22]. Here we present metagenomic data for MTB from diverse natural environments from both the northern and the southern hemispheres. Comparison and analyses of these reconstructed genomes provide great insight into the phylogenetic diversity of MTB and the origin and evolution of magnetotaxis as well as in iron-based biomineralization on Earth.

## Materials and Methods

### Sample collection and MTB characterization

Surface sediments were collected from 13 locations from aquatic areas in China and Australia (Supplementary Table 1 and Supplementary Figure 1a). The collected sediments were transferred to flasks, transported to the laboratory, and incubated at room temperature without disturbance. MTB cells were enriched magnetically using a “MTB trap” [23]. The collected cells were washed and resuspended in sterile distilled H_2_O. The morphologies of MTB cells were analyzed and characterized as described previously [24] using a JEM-2100HR transmission electron microscope operated at 200 kV, with energy dispersive spectroscopy (Oxford X-Max 80).

### Metagenomic sequencing, scaffold assembly, and genome binning

Metagenomic DNA was extracted and amplified from magnetically enriched MTB cells as previously described [13]. Shotgun sequencing of metagenomic DNA from each location was performed with an Illumina HiSeq 2000 using the pair-end 2 × 125 reads with a 600-bp insert size or using the Illumina HiSeq 4000 with the pair-end strategy of 150-bp reads with an average 270-bp insert size (Beijing Genomics Institute, Beijing, China). Illumina reads were trimmed to remove the adapter sequences and low-quality bases, and were assembled using metaSPAdes [25] with the following parameters (--only-assembler -k 31,41,51,61,71,81,91,101,111,121). Assembled scaffolds ≥ 2500 bp were binned separately using MetaBAT v0.26.1 [26] and MyCC [27]. Results of two binning methods for each sample were combined and a non-redundant set of bins was chosen. The acquired genomes were curated manually with two approaches: (1) using the CheckM [28] “outliers” command to identify scaffolds from bins that appear to be outliers in either GC, tetranucleotide, or coding density space relative to the expected distribution of these genomic statistics; and (2) using BLASTn or BLASTx to identify potential contaminant contigs based on their top BLAST hits. The quality and accuracy of the acquired genomes were assessed using CheckM [28] based on the taxonomic-specific workflow (domain *Bacteria*) and QUAST [29]. Genomes were annotated using Prokka v1.11 [30] with manual curation. Candidate magnetosome genes were checked manually and verified using the NCBI BLAST webserver [31]. The average amino-acid identity (AAI) was calculated using enveomics [32].

### Phylogenetic analyses

**Fig. 1:**
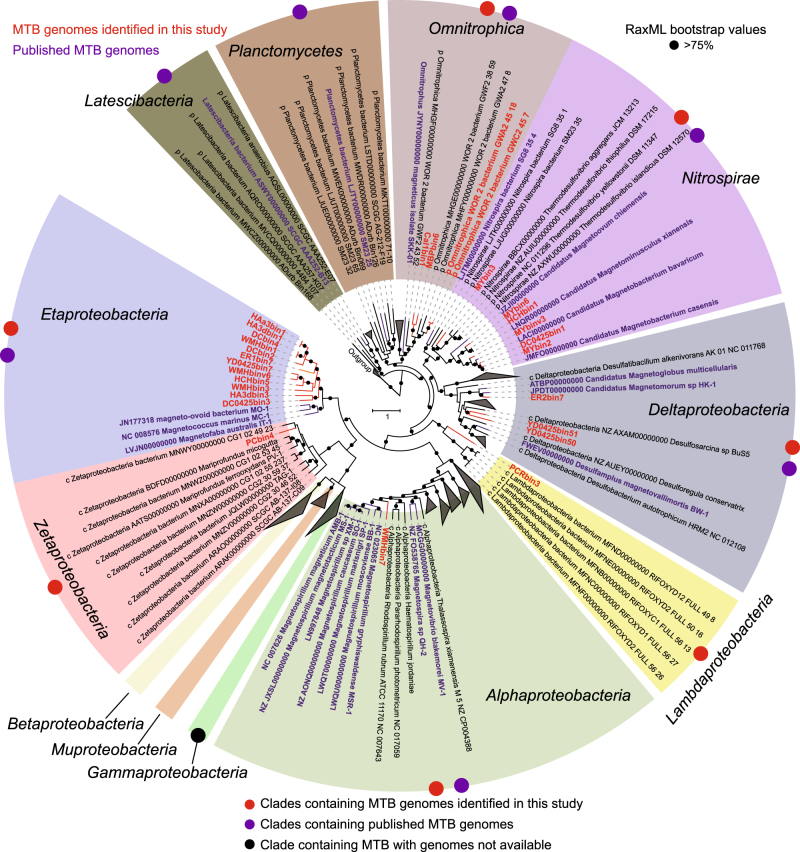
Maximum likelihood phylogeny of MTB genomes. Phylogenomic tree based on concatenated alignment (3973 amino-acid positions) of up to 400 ubiquitous conserved proteins identified with PhyloPhlAn [34]. Archaeal genomes (accession numbers CP001719 and CP000678) were used as the outgroup. Red and purple denote MTB genomes from this study and from published MTB genome sequences, respectively. Bootstrap values are indicated with black circles (>75% support from 100 resamples). Detailed characteristics of genome sequences recovered in this study are summarized in Supplementary Table 2 (color figure online)

The maximum likelihood phylogeny of genomes was constructed using RAxML v8.2.8 [33] (-m PROTGAMMAVT -f a -x 12345 -k -p 12345 -N 100) with a concatenated alignment of the conserved ubiquitous proteins identified with PhyloPhlAn [34] (Fig. 1 and Supplementary Figure 2). The VT+G model was used as determined by ProtTest 3.4 [35]. The genomic tree was rooted with genomes from the domain *Archaea* (*Methanobrevibacter ruminantium* and *Methanobrevibacter smithii*). Confidence in phylogenetic results was assessed using the rapid bootstrap algorithm of RAxML with 100 replicates [36]. Bootstrap convergence test was conducted using RAxML (-I autoMRE). In order to further identify whether the *Magnetococcales* order represent a novel class in the *Proteobacteria* phylum, we additionally constructed a maximum likelihood phylogenomic tree with a concatenated amino-acid sequence alignment (6988 amino-acid positions) of 43 lineage-specific marker genes from 15 *Magnetococcales* genomes and up to 248 *Proteobacteria* genomes generated using the “tree” command in CheckM [28] (Supplementary Figure 3). The genomic tree was constructed using RAxML v8.2.8 [33] under the LG+I+G model of evolution.

Homologous sequences of magnetosome proteins MamA, -B, -E, -K, -M, and -Q were identified within the refseq_protein database using PSI-BLAST searches (BLOSUM62 scoring matrix, *E*-value < 1e-05, with exclusion of published MTB genomes) with each magnetosome protein from *Magnetospirillum gryphiswaldense* MSR-1, *Desulfovibrio magneticus* RS-1, and “*Candidatus* Magnetoglobus multicellularis” as query sequences. The hits were combined and clustered using CD-HIT [37] with a sequence similarity cutoff of 0.8. The complete amino-acid sequences of magnetosome proteins MamA, -B, -E, -K, -M, and -Q from all available MTB genomes and their non-MTB homologs were aligned by MUSCLE [38] algorithms using MEGA v6.06 [39]. Phylogenetic trees were then generated using the maximum likelihood method of FastTree v2.1.9 [40] with default settings. Multiple alignments of MamE, -M, and -Q were concatenated and a phylogenetic tree was constructed using RAxML v8.2.8 [33] with the LG+I+G model as determined by ProtTest 3.4 [35]. Confidence in a phylogenetic tree was assessed using 100 bootstrap replicates. Trees were visualized using FigTree v1.4.2 (http://tree.bio.ed.ac.uk/software/figtree/) and iTOL [41].

### Data availability

Genome sequences have been deposited in the NCBI BioProject (Magnetotactic Bacteria Metagenome Project (MagMeta)) under accession number PRJNA400260 with genome accession numbers PDZS00000000–PEAR00000000.

## Results and Discussion

### Metagenome-assembled MTB genomes

To obtain a large number of MTB genomes and to better understand their genomic diversity and evolution, we conducted, to the best of our knowledge, the first large-scale metagenomic survey of MTB from varied aquatic environments, including lakes, ponds, rivers, rice fields, and creeks, in both the northern and the southern hemispheres (Supplementary Table 1 and Supplementary Figure 1a). Various MTB morphotypes, including cocci, rods, vibrios, and spirilla that contain iron-oxygen and/or iron-sulfur magnetosomes were identified in samples collected from these environments (Supplementary Figure 1b and c).

Metagenomic DNA sequences were extracted from magnetically enriched MTB cells and were sequenced, assembled, and binned to draft genomes. A total of 26 high-quality MTB genomes were reconstructed (67–98% completeness with 90% average; Supplementary Table 2). These new genome sequences span diverse taxonomic lineages: 18 affiliated with the phylum *Proteobacteria*, 6 with the phylum *Nitrospirae*, and 2 with the phylum *Omnitrophica* (Fig. 1). We also included two published genomes from the *Omnitrophica* phylum [42] from public databases containing nearly complete MGCs (Supplementary Table 2). These MGCs were previously unrecognized and were identified here, resulting in total 28 novel MTB genomes in this study (Fig. 1). Most of these new genomes (24 of 28) are phylogenetically divergent from previously known MTB genomes emphasizing the great phylogenetic diversity of MTB.

### Phylogenomic inference

**Fig. 2:**
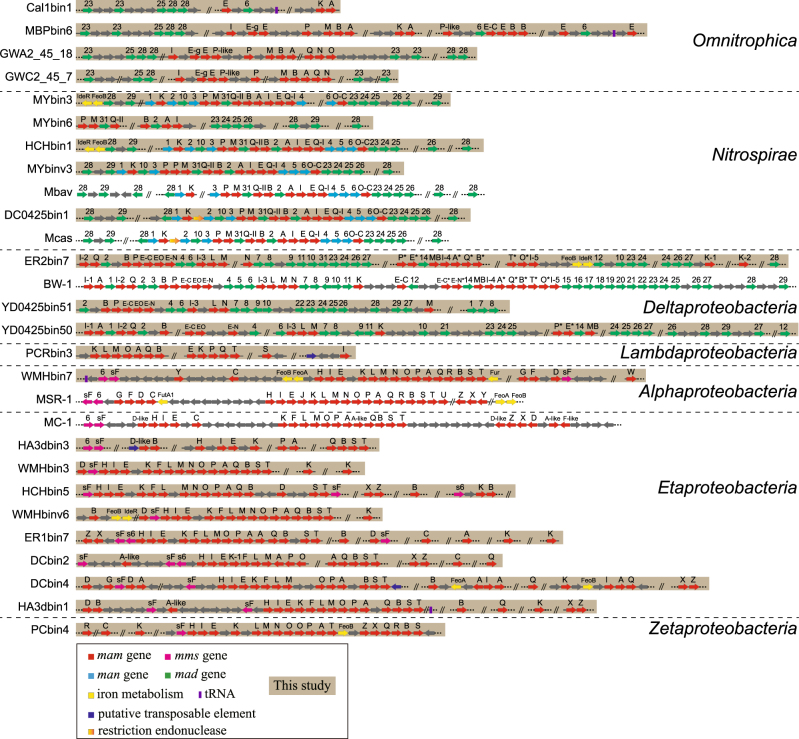
Representative magnetosome gene clusters (MGCs) in MTB genomes. Comparison of representative MGCs recovered in this study with previously identified representative MGCs. Mbav “*Candidatus* Magnetobacterium bavaricum”, Mcas “*Candidatus* Magnetobacterium casensis”, BW-1 *Desulfamplus magnetovallimortis* BW-1, MSR-1 *Magnetospirillum gryphiswaldense* MSR-1, MC-1 *Magnetococcus marinus* MC-1

Whether classes in the *Proteobacteria* phylum other than the *Alphaproteobacteria*, *Deltaproteobacteria*, and *Gammaproteobacteria* contain MTB is a key question in understanding the evolution of magnetotaxis in this phylum [15]. A striking finding of our study is the affiliation of two MTB genomes within the *Zetaproteobacteria* (PCbin4) and “*Candidatus* Lambdaproteobacteria” (PCRbin3) classes, respectively, in the *Proteobacteria* phylum (Fig. 1). The *Zetaproteobacteria* recognized so far are neutrophilic, lithotrophic marine Fe^2+^-oxidizing bacteria commonly found in Fe^2+^-rich environments from hydrothermal vents to coastal environments [43, 44], although members of this group have been recently identified in marine environments that do not contain elevated concentrations of Fe [45]. PCbin4 contains a nearly complete Fe_3_O_4_-type MGC (Fig. 2). Considering that all currently known isolates of *Zetaproteobacteria* are obligate microaerophilic, lithotrophic Fe^2+^-oxidizing bacteria, our results might indicate that PCbin4 may be capable of biomineralizing both intracellular and extracellular iron minerals, although further investigation is necessary.

“*Candidatus* Lambdaproteobacteria” is a candidate class recently proposed based on metagenome-assembled genome sequences [42]. There are currently no cultivated representatives of this class and little is known regarding their physiology. PCRbin3 clusters robustly within the “*Candidatus* Lambdaproteobacteria” (100 bootstrap value) and represents the earliest diverging clade in this class (Fig. 1). The first findings of MTB from the *Zetaproteobacteria* and “*Candidatus* Lambdaproteobacteria” classes extend the phylogenetic diversity of MTB in the *Proteobacteria* phylum and further indicate that magnetotaxis is widespread in this phylum.

Previous cultivation-independent surveys indicate the presence of large MTB populations phylogenetically affiliated with the order *Magnetococcales* from both freshwater and marine habitats resulting in these organisms being considered a or the dominant MTB group in many environments [46]. Despite their widespread distribution, only three marine strains have been isolated in axenic cultures and have had their genomes sequenced, which makes the phylogenetic placement of the *Magnetococcales* inconclusive [47–49]. rRNA gene-based analyses indicate an affiliation of *Magnetococcales* within the *Alphaproteobacteria* class as the deepest-diverging branch [50] or even as a subclass [51]. Comparative genomic studies, however, suggest a high level of mosaic origins of *Magnetococcales* genomes [52] and it has been suggested recently that this order represents a new class (i.e., “*Candidatus* Etaproteobacteria”) in the phylum *Proteobacteria* [47]. We reconstructed 12 *Magnetococcales* genomes here, which substantially expand the genomic representation of this group. To explore more accurately the phylogenetic placement of the *Magnetococcales*, in addition to the genomic tree constructed from concatenated conserved ubiquitous proteins using PhyloPhlAn [34] (Fig. 1 and Supplementary Figure 2), we constructed a phylogenomic tree based on a concatenated alignment of amino-acid sequences of marker genes identified by CheckM [28] (Supplementary Figure 3). The exact position of the *Magnetococcales* in two genomic trees is not consistent: in the PhyloPhlAn tree the *Magnetococcales* represents a sister clade to the classes of *Betaproteobacteria*, *Gammaproteobacteria*, *Zetaproteobacteria*, and “*Candidatus* Muproteobacteria” (Fig. 1), while in the CheckM tree, *Magnetococcales* is a sister clade to the *Alphaproteobacteria* (Supplementary Figure 3). Despite these inconsistencies, both trees provide strong support for *Magnetococcales* as a novel monophyletic class of *Proteobacteria* (i.e., “*Candidatus* Etaproteobacteria”), rather than as an order within the *Alphaproteobacteria*. The exact phylogenetic placement of “*Candidatus* Etaproteobacteria” relative to other classes in the *Proteobacteria* phylum awaits further investigation when more genomic sequences become available.

One (WMHbin7) and three (ER2bin7, YD0425bin50, and YD0425bin51) genomes were identified as belonging to the *Alphaproteobacteria* and *Deltaproteobacteria* classes, respectively (Fig. 1). WMHbin7 forms a distinct lineage in the order *Rhodospirillales* and represents a sister group to the well-characterized genus *Magnetospirillum* (Fig. 1). ER2bin7 is phylogenetically closely related to the uncultured, multicellular magnetotactic prokaryotes (MMPs) “*Candidatus* Magnetomorum” sp. HK-1 (HK-1) [53] and “*Candidatus* Magnetoglobus multicellularis” (MMP) [54]. The average AAI value between ER2bin7 and HK-1 is 74%, which is higher than the genus criterion level of 65% [55], indicates that ER2bin7 and HK-1 belong to the same genus. Similar to HK-1, two sets of putative Fe_3_O_4_- and Fe_3_S_4_-type magnetosome genes were identified in ER2bin7, which suggests that this organism biomineralizes both types of magnetosomes in the same types of cells (Fig. 2). Supporting this suggestion is the discovery of a population of MTB cells from sample ER2 that contain both iron-oxygen and iron-sulfur magnetosomes (Supplementary Figure 1c). The AAI value between YD0425bin50 and YD0425bin51 is only 53%, which indicates that they represent organisms of two different genera. Both Fe_3_O_4_- and Fe_3_S_4_-type magnetosome genes were identified in YD0425bin50, while only one MGC with high similarity to Fe_3_O_4_-type magnetosome genes was found in YD0425bin51 (Fig. 2). YD0425bin50 and YD0425bin51 have only a distant phylogenetic relationship to other MTB of the *Deltaproteobacteria*, including ER2bin7, HK-1, MMP, and *Desulfamplus magnetovallimortis* BW-1 (Fig. 1), which suggests that there is considerable diversity of MTB in the *Deltaproteobacteria* remaining to be described. Further studies, such as 16S rRNA gene-based identification and cultivation-dependent analysis, will provide a deeper insight into the diversity of magnetotactic *Deltaproteobacteria*.

MTB in the phylum *Nitrospirae* are of interest because some members of this phylum synthesize hundreds of intracellular bullet-shaped Fe_3_O_4_ magnetosomes and large amounts of sulfur granules [56], which suggests that they contribute significantly to iron and sulfur cycling in natural environments. We obtained six genomes that are affiliated phylogenetically with this phylum that belong clearly to two distinct clusters: one consisting of previously reported MTB of this phylum, while MYbin3, a new genus according to the low AAI values (<60%) with respect to other genomes, is affiliated with another group that is related distantly to the genus *Thermodesulfovibrio* (Fig. 1). Considering that a thermophilic population of MTB distantly related to the *Thermodesulfovibrio* was identified from hot springs [57] and recent identification of a putative MTB genome (Nitrospira bacterium SG8_35_4) from another cluster (Fig. 1) [16], MTB of the phylum *Nitrospirae* are very likely more diverse than previously thought. In agreement with previous studies [12, 13], the gene content and order of MGCs from the *Nitrospirae* were highly conserved despite the wide phylogenetic distance between organisms (Fig. 2). HCHbin1 has an average AAI value of >99% with the genome of “*Candidatus* Magnetominusculus xianensis” strain HCH-1 recovered from the same sample using a different assembly and binning approach from our previous study [13], which indicates that these two genomes are from the same organism. Similarly, the genomes of MYbin2 and “*Candidatus* Magnetobacterium casensis” [12] appear to be from the same organism (AAI value > 98%).

MTB in the candidate phylum *Omnitrophica* were discovered only recently and so far only one population with a draft genome containing five scattered magnetosome genes has been identified [8]. Whether MTB in this phylum contain a MGC and, if so, how it is organized remains unclear. We recovered two MTB genomes (Cal1bin1 and MBPbin6) phylogenetically affiliated with this phylum. We also identified two published *Omnitrophica* genomes (Omnitrophica WOR_2 bacterium GWC2_45_7 and Omnitrophica WOR_2 bacterium GWA2_45_18) both containing nearly complete MGCs from the GenBank database (Fig. 2). These four genomes harbor MGCs with high similarity both in gene content and order. These MGCs, however, appear to be distinct from known MGCs from MTB of other phyla, which suggests that a hidden reservoir of MGC variants exists in previously unknown MTB lineages (Fig. 2).

### Characterization of MGCs

The new genomes acquired in this study contain partial or nearly complete MGCs with genes homologous to magnetosome genes *mam*, *mms*, *mad*, and *man*, providing an opportunity to address fundamental issues concerning the origin and evolution of magnetotaxis (Fig. 2). In general, magnetosome gene organization in each MGC corresponds with their taxonomies, that is, MGCs were more conserved in terms of gene content and organization within closely related groups than those between different taxonomic lineages (Fig. 2). The overall structures of MGCs from the phylum *Proteobacteria* (without the class *Deltaproteobacteria*) appear to be different from those of deep-branching MTB belonging to the class *Deltaproteobacteria*, the phylum *Nitrospirae*, and the phylum *Omnitrophica*. *mam* genes are present in all acquired MGCs, while only MGCs from deep-branching MTB contain *mad* and *man* genes. Specifically, *mad* genes have been identified in magnetotactic *Deltaproteobacteria*, *Nitrospirae*, and *Omnitrophica*, while *man* genes have only been found in *Nitrospirae* MTB (Fig. 2).

Despite general conservation, it appears that the gene order and abundance of MGCs in each class of the *Proteobacteria* are less conserved than those of the *Nitrospirae* and *Omnitrophica* (Fig. 2). For example, multiple copies of *mamK* genes have been identified within several MGCs of “*Candidatus* Etaproteobacteria” (e.g., WMHbin3, HCHbin5, WMHbinv6, ER1bin7, DCbin4, and HA3dbin1), but not in the other MGCs of this class, and MGCs of ER2bin7 and YD0425bin50 in the *Deltaproteobacteria* have considerable variability in the gene content and order (Fig. 2). One possible explanation is that this heterogeneity may be attributed to the incomplete nature of MGCs recovered through the metagenome-assembled method and/or the artificial displaying order of scaffolds for each MGC. Conversely, this variability may represent the true nature of MGCs in these MTB. The heterogeneity of MGCs has been noted previously in axenic cultures of “*Candidatus* Etaproteobacteria” [47, 49, 58] and within the magnetotactic *Deltaproteobacteria* [10]. Two copies of *mamK* genes have been found within MGCs of some cultivated MTB strains, such as *Magnetovibrio blakemorei* MV-1 and magnetotactic *Gammaproteobacteria* strain SS-5 [10, 11]. Genetic events, such as gene duplication, rearrangement, acquisition, and loss, may contribute to variability of MGCs, and account for the large diversity in the morphology, number, and arrangement of magnetosomes observed in MTB.

### Origin and evolution of magnetotaxis

**Fig. 3:**
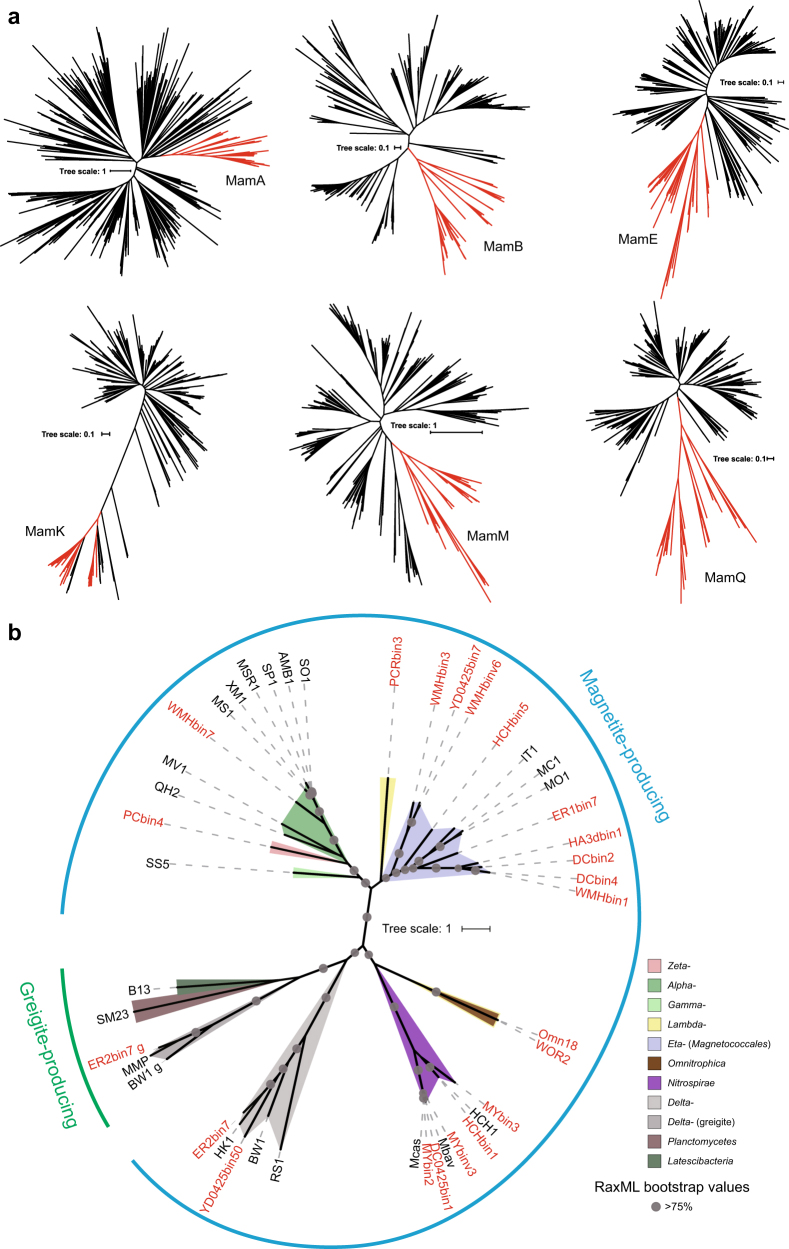
Phylogenetic analyses of core magnetosome proteins. **a** Phylogenies of magnetosome proteins MamA, -B, -E, -K, -M, and -Q (red) and their non-MTB homologs (black). **b** Maximum-likelihood phylogenetic tree based on concatenated protein alignment of MamEMQ. Sequences identified in this study are shown in red. AMB1 *Magnetospirillum magneticum* AMB-1, MSR-1 *Magnetospirillum gryphiswaldense* MSR-1, MS-1 *Magnetospirillum magnetotacticum* MS-1, XM-1 *Magnetospirillum* sp. XM-1, SO-1 *Magnetospirillum caucaseum* SO-1, SP-1 *Magnetospirillum marisnigri* SP-1, MV-1 *Magnetovibrio blakemorei* MV-1, QH-2 *Magnetospira* sp. QH-2, SS-5 *Gammaproteobacteria* bacterium strain SS-5, IT-1 *Magnetofaba australis* IT-1, MC-1 *Magnetococcus marinus* MC-1, MO1 “*Candidatus* Magnetococcus massalia”, HK-1 “*Candidatus* Magnetomorum” sp. HK-1, BW-1 *Desulfamplus magnetovallimortis* BW-1, RS-1 *Desulfovibrio magneticus* RS-1, MMP “*Candidatus* Magnetoglobus multicellularis”, Mbav “*Candidatus* Magnetobacterium bavaricum”, Mcas “*Candidatus* Magnetobacterium casensis”, HCH-1 “*Candidatus* Magnetominusculus xianensis” strain HCH-1, B13 *Latescibacteria* bacterium SCGC AAA252-B13, SM23 *Planctomycetes* bacterium SM23_25 (color figure online)

To examine the evolutionary history of magnetotaxis, amino-acid sequences of core magnetosome proteins MamA, -B, -E, -K, -M, and -Q were used to identify homologs across GenBank. For each of six protein phylogenies, magnetosome proteins all grouped together and, with the exception of MamK, the rest all form a well-supported monophyletic clade to the exclusion of non-MTB homologs (Fig. 3a). These results support the idea that both Fe_3_O_4_- and Fe_3_S_4_-type core magnetosome genes across different taxa have a common origin. The identification of MamK, a protein involved in the construction of magnetosome chains, in the genomes of non-MTB has been previously noted [10]. It has been proposed that *mamK* genes in the genomes of non-MTB are either acquired through horizontal gene transfer (HGT) from MTB or represent MGC relics [10]. The phylogeny based on concatenated magnetosome proteins is largely in agreement with bacterial systematics, which represents the monophyly of major classes or phyla (Fig. 3b). In addition, only a small group of acquired MGCs contain putative transposable elements and tRNA genes (Fig. 2). These results indicate that recent HGT between classes or phyla was rare during the evolution of magnetotaxis and that the origin of magnetotaxis and magnetosome biomineralization is ancient [13, 59]. The only exceptions are the Fe_3_S_4_-type MGCs from the *Deltaproteobacteria* class, which, although distantly related, group with those from the phyla *Latescibacteria* and *Planctomycetes* (Fig. 3b).

**Fig. 4:**
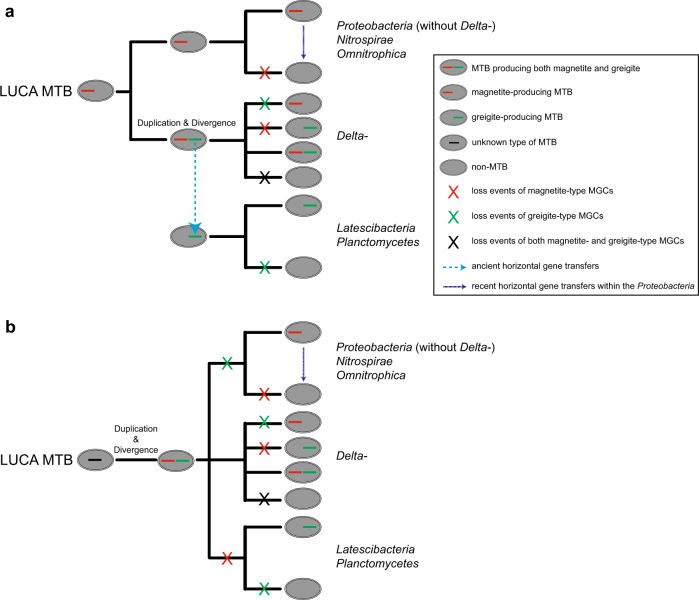
Two models for the evolution of magnetotaxis in the domain *Bacteria*. **a** The last universal common ancestor of magnetotactic bacteria (LUCA MTB) was a magnetite (Fe_3_O_4_)-producing MTB present as the ancestor of each of the *Proteobacteria*, *Nitrospirae*, and *Omnitrophica* phyla. Multiple independent instances of MGC loss then ensued in each phylum or class. The Fe_3_O_4_-type MGC was duplicated and one diversified to a greigite (Fe_3_S_4_)-type MGC in the *Deltaproteobacteria*. The Fe_3_S_4_-type MGC is hypothesized to have been transferred to *Latescibacteria* and *Planctomycetes*. These transfer events must have occurred early because of the large phylogenetic distance between Fe_3_S_4_ magnetosome proteins of *Deltaproteobacteria* and the *Latescibacteria* and *Planctomycetes* phyla (Fig. 3b). **b** In the second hypothesis the LUCA MTB contained an unknown type of ancient MGC, which was duplicated and diverged to generate both Fe_3_O_4_- and Fe_3_S_4_-type MGCs in the last common ancestor of the *Proteobacteria*, *Nitrospirae*, *Omnitrophica*, *Latescibacteria*, and *Planctomycetes* phyla. Multiple instances of MGC loss then occurred during evolution, with some lineages losing both clusters while others retained one or two types. For both hypothetical scenarios, vertical inheritance followed by multiple independent MGC losses is considered to be the major force that drove evolution of magnetotaxis, although recent horizontal transfers of MGCs might have occurred within some classes, genera (e.g., *Magnetospirillum*), or species

These new data allow us to propose that the core magnetosome genes, at least for Fe_3_O_4_-type genes, were present in the ancestor of each of the *Proteobacteria*, *Nitrospirae*, and *Omnitrophica* phyla (Fig. 4a) or in the last common ancestor of the *Proteobacteria*, *Nitrospirae*, *Omnitrophica*, *Latescibacteria*, and *Planctomycetes* phyla (Fig. 4b). The subsequent evolutionary history of MGCs in each taxonomic lineage then diversified. For Fe_3_O_4_-producing MTB, vertical inheritance followed by multiple independent losses of MGCs is likely the major force that drove the evolution of magnetotaxis, although potential recent HGT of MGCs has been suggested between some MTB within the same class or genus (e.g., *Magnetospirillum*) in the *Proteobacteria* phylum [47, 60]. Due to the limited studies of Fe_3_S_4_-producing MTB, the origin and evolution of this type of MGC are poorly understood. *Deltaproteobacteria* MTB were the only known group containing both Fe_3_O_4_- and Fe_3_S_4_-type MGCs in the same genome, so it has previously been proposed that Fe_3_S_4_ magnetosome formation originated in this class through duplication and subsequent divergence of Fe_3_O_4_-type MGCs [15]. Owing to the large phylogenetic distance between magnetosome proteins of *Deltaproteobacteria* and of phyla *Latescibacteria* and *Planctomycetes* (Fig. 3b), we suggest that the duplication and divergence event occurred early in the *Deltaproteobacteria* and that MGCs in the phyla *Latescibacteria* and *Planctomycetes* were acquired through ancient HGT from the *Deltaproteobacteria* during evolution (Fig. 4a). Alternatively, we cannot exclude that duplication and divergence of ancient unknown MGC types generated both Fe_3_O_4_- and Fe_3_S_4_-type MGCs in the last common ancestor of the *Proteobacteria*, *Nitrospirae*, *Omnitrophica*, *Latescibacteria*, and *Planctomycetes* phyla (Fig. 4b). We propose that as the phyla diversified, multiple instances of MGC loss occurred during evolution, with some lineages losing both clusters and others retaining one or both types (Fig. 4b).

The processes by which MGCs were lost multiple times across different phyla remain to be understood. The biosynthetic Fe_3_O_4_ magnetosome pathway is complex and has been shown to be composed of >30 genes and >100 kb section of DNA sequence in the *Magnetospirillum* [3]. The frequent spontaneous loss of magnetosome genes in many cultivated MTB strains has been noted [61], which suggests that the metabolic cost of replicating MGCs and forming magnetosomes is high. Thus, in nature, the selective pressure driving MGC maintenance would need to be strong otherwise microorganisms would be expected to lose MGCs, thereby losing the capability of magnetosome formation when the biological advantage of magnetotaxis is small [59]. Further metagenomic and genomic sequencing of MTB from different phylogenetic lineages will undoubtedly provide valuable insights into the processes by which MTB maintained and lost MGCs.

Our work not only illustrates the unexpectedly large phylogenetic diversity of MTB in nature but also illustrates some of the possible evolutionary routes of magnetotaxis. It seems clear that magnetotaxis in the domain *Bacteria* is an ancient physiological trait that has a single common origin with lineage-specific evolution (i.e., it followed different evolutionary routes in different taxonomic lineages). The evolutionary history of magnetotaxis might be complex, with vertical inheritance followed by independent lineage losses as the major evolutionary force. Although the origin and evolution of Fe_3_S_4_-type magnetotaxis needs further investigation, our analysis also suggests an early origin of Fe_3_S_4_ magnetosomes in bacteria (Fig. 4). Detailed examination of novel MTB genomes, together with 16S rRNA gene- or magnetosome gene-based fluorescence *in situ* hybridization and electron microscopy studies, will further our understanding of morphological diversity, ecophysiology, and metabolic potential of these poorly characterized MTB. It should not be surprising if future work reveals new MTB with novel types of MGCs in other, as yet undiscovered, lineages across these five phyla or even across other Bacterial phyla.

## Electronic supplementary material

Supplementary Information(DOCX 125 kb)

Supplementary Table 1(DOCX 98 kb)

Supplementary Table 2(DOCX 99 kb)

Supplementary Figure 1(JPG 4220 kb)

Supplementary Figure 2(PDF 1650 kb)

Supplementary Figure 3(PDF 544 kb)
