# Supplementary material for: Genomic expansion of magnetotactic bacteria reveals an early common origin of magnetotaxis with lineage-specific evolution
Source: ISME J. 2018 Mar 26;12(6):1508–19. doi: 10.1038/s41396-018-0098-9 (PMC5955933; doi:10.1038/s41396-018-0098-9)
Supplement: Supplementary file 1 — Supplementary Information(DOCX 125 kb) [file 41396_2018_98_MOESM1_ESM.docx]

**Supplementary Information**

**Genomic expansion of magnetotactic bacteria reveals an early common origin of magnetotaxis with lineage-specific evolution**

Wei Lin^1,2,3*^, Wensi Zhang^1,2,3,4^, Xiang Zhao^5^, Andrew P. Roberts^5^, Greig A. Paterson^1,2,6^, Dennis A. Bazylinski^7^, Yongxin Pan^1,2,3,4*^

^1^Key Laboratory of Earth and Planetary Physics, Institute of Geology and Geophysics, Chinese Academy of Sciences, Beijing, 100029, China.

^2^Institutions of Earth Science, Chinese Academy of Sciences, Beijing, 100029, China

^3^France-China Joint Laboratory for Evolution and Development of Magnetotactic Multicellular Organisms, Chinese Academy of Sciences, Beijing, 100029, China.

^4^College of Earth Sciences, University of Chinese Academy of Sciences, Beijing, 100049, China.

^5^Research School of Earth Sciences, Australian National University, Canberra, ACT 2601, Australia.

^6^Department of Earth, Ocean and Ecological Sciences, University of Liverpool, Liverpool, L69 7ZE, UK.

^7^School of Life Sciences, University of Nevada at Las Vegas, Las Vegas, NV 89154-4004, USA.

*E-mails: [weilin0408@gmail.com](mailto:weilin0408@gmail.com) (W.L.) or [yxpan@mail.iggcas.ac.cn](mailto:yxpan@mail.iggcas.ac.cn) (Y.P.)

**Running title:** Origin and evolution of magnetotaxis

**Subject Categories:** Integrated genomics and post-genomics approaches in

microbial ecology

**Supplementary Figure 1** Sampling sites and transmission electron microscope (TEM) images of representative MTB cells. (**a**) Location of the 13 sampling sites in China and Australia (the map was generated using the GeoMapApp 3.6.0; http://www.geomapapp.org/). Further site details are given in Supplementary Table 1. (**b**) Various morphotypes of MTB cells with different magnetosome types. (**c**) TEM image, with both corresponding energy-dispersive X-ray spectra, of MTB from Erskine River (ER2), Australia, with both iron-oxygen and iron-sulphur magnetosomes.

**Supplementary Figure 2** Full linear view of the phylogenetic genomic tree presented in Figure 1, based on concatenated amino acid sequences of marker gene alignment generated using PhyloPhlAn. Red and purple denote MTB genomes identified in this study and from published MTB genome sequences, respectively. Bootstrap support values above 75% are indicated by grey circles on the nodes.

**Supplementary Figure 3** Phylogenetic genomic tree based on concatenated amino acid sequences of marker gene alignment generated using CheckM showing the placement of the “*Candidatus* Etaproteobacteria” (*Magnetococcales*) in the *Proteobacteria* phylum. Red and purple denote MTB genomes identified in this study and from published MTB genome sequences, respectively. *Latescibacteria* genomes were used as the outgroup. Bootstrap support values above 75% are indicated by grey circles on the nodes.

**Supplementary Table 1** Overview of the sample sites in this study.

**Supplementary Table 2** General characteristics of the 28 MTB genomes reported in this study.
