# Supplementary material for: Genomic expansion of magnetotactic bacteria reveals an early common origin of magnetotaxis with lineage-specific evolution
Source: ISME J. 2018 Mar 26;12(6):1508–19. doi: 10.1038/s41396-018-0098-9 (PMC5955933; doi:10.1038/s41396-018-0098-9)
Supplement: Supplementary file 2 — Supplementary Table 1(DOCX 98 kb) [file 41396_2018_98_MOESM2_ESM.docx]

**Supplementary Table 1** Overview of the sample sites in this study

| **Sample ID** | **Sample location** | **Latitude (°)** | **Longitude (°E)** | **Salinity (ppt)** | **pH** | **Temperature (°C)** |
| --- | --- | --- | --- | --- | --- | --- |
| Cal1 | Lake Catani, Australia | -36.73426 | 146.81119 | < 0.1 | / | 12 |
| DC | Lake Dianchi, China | 24.908133 | 102.74605 | 0.9 | 7.6 | / |
| ER1 | Erskine River, Australia | -38.5338 | 143.97832 | 3.2 | / | 14 |
| ER2 | Erskine River, Australia | -38.5359 | 143.97478 | 1.9 | / | 12 |
| HA3d | Pond, Hongan, China | 31.173819 | 114.547642 | 0.2 | 5.2 | / |
| HAa3 | Rice field, Hongan, China | 31.174289 | 114.543941 | < 0.1 | 5.2 | / |
| HCH | Xi’an city moat, China | 34.25287 | 108.92187 | 0.2 | 7.5 | / |
| MBP | Mount Beauty Pondage, Australia | -36.73874 | 147.16443 | < 0.1 | / | 14 |
| MY | Lake Miyun, China | 40.48874 | 117.00714 | 0.2 | 7.5 | / |
| PC | Painkalac Creek, Australia | -38.46575 | 144.09288 | 21.7 | / | 17 |
| PCR | Punkally Creek, Australia | -36.23413 | 150.06798 | 33.9 | / | 25 |
| WMH | Lake Weiming, China | 39.993142 | 116.30256 | 0.3 | 7.5 | / |
| YD0425 | Yuandadu Park, China | 39.974732 | 116.368688 | 0.5 | 7.2 | / |
