# Supplementary material for: Genomic expansion of magnetotactic bacteria reveals an early common origin of magnetotaxis with lineage-specific evolution
Source: ISME J. 2018 Mar 26;12(6):1508–19. doi: 10.1038/s41396-018-0098-9 (PMC5955933; doi:10.1038/s41396-018-0098-9)
Supplement: Supplementary file 3 — Supplementary Table 2(DOCX 99 kb) [file 41396_2018_98_MOESM3_ESM.docx]

**Supplementary Table 2** General characteristics of the 28 MTB genomes reported in this study.

| **MTB genome** | **Accession no.** | **Taxonomy** | **Size (bp)** | **Scaffolds (no.)** | **GC (%)** | **N50 (bp)** | **Estimated completeness (%)^*^** | **Estimated contamination (%)^*^** | **Location** | **Reference** |
| --- | --- | --- | --- | --- | --- | --- | --- | --- | --- | --- |
| Cal1bin1 | PEAR00000000 | *Omnitrophica* | 2540141 | 240 | 49.55 | 15133 | 89.66 | 1.72 | Lake Catani, Australia | This study |
| DCbin2 | PEAO00000000 | *Proteobacteria*; *Etaproteobacteria* | 3364396 | 201 | 51.85 | 24837 | 74.14 | 0 | Lake Dianchi, China | This study |
| DCbin4 | PEAN00000000 | *Proteobacteria*; *Etaproteobacteria* | 4521331 | 148 | 54.25 | 49732 | 91.38 | 0 | Lake Dianchi, China | This study |
| DC0425bin1 | PEAQ00000000 | *Nitrospirae* | 4024796 | 107 | 49.04 | 75024 | 96.55 | 0 | Lake Dianchi, China | This study |
| DC0425bin3 | PEAP00000000 | *Proteobacteria*; *Etaproteobacteria* | 3696641 | 230 | 65.4 | 26687 | 98.28 | 0 | Lake Dianchi, China | This study |
| ER1bin7 | PEAM00000000 | *Proteobacteria*; *Etaproteobacteri* | 3866744 | 92 | 52.3 | 116043 | 98.28 | 0 | Erskine River, Australia | This study |
| ER2bin7 | PEAL00000000 | *Proteobacteria*; *Deltaproteobacteira* | 5965504 | 653 | 38.47 | 12662 | 91.38 | 1.88 | Erskine River, Australia | This study |
| HA3dbin1 | PEAK00000000 | *Proteobacteria*; *Etaproteobacteria* | 4326896 | 134 | 53.32 | 54627 | 98.12 | 0 | Pond, Hongan, China | This study |
| HA3dbin3 | PEAJ00000000 | *Proteobacteria*; *Etaproteobacteria* | 2895712 | 316 | 61.72 | 13187 | 70.69 | 0 | Pond, Hongan, China | This study |
| HAa3bin1 | PEAI00000000 | *Proteobacteria*; *Etaproteobacteria* | 4347394 | 118 | 53.21 | 63208 | 98.12 | 0 | Rice field, Hongan, China | This study |
| HCHbin1 | PEAH00000000 | *Nitrospirae* | 3692324 | 94 | 45.24 | 55039 | 96.55 | 0 | Xi’an city moat, China | This study |
| HCHbin5 | PEAG00000000 | *Proteobacteria*; *Etaproteobacteria* | 4188618 | 200 | 56.97 | 38237 | 98.28 | 0.69 | Xi’an city moat, China | This study |
| MBPbin6 | PEAF00000000 | *Omnitrophica* | 2181257 | 150 | 49.49 | 24179 | 67.08 | 1.72 | Mount Beauty Pondage, Australia | This study |
| MYbin2 | PEAE00000000 | *Nitrospirae* | 3071492 | 89 | 48.79 | 62390 | 75.86 | 0 | Lake Miyun, China | This study |
| MYbin3 | PEAD00000000 | *Nitrospirae* | 2929370 | 66 | 44.36 | 86724 | 92.01 | 0 | Lake Miyun, China | This study |
| MYbin6 | PEAC00000000 | *Nitrospirae* | 3596763 | 218 | 47.78 | 26590 | 76.96 | 0 | Lake Miyun, China | This study |
| MYbinv3 | PEAB00000000 | *Nitrospirae* | 3713224 | 175 | 44.44 | 35197 | 93.97 | 1.72 | Lake Miyun, China | This study |
| PCbin4 | PEAA00000000 | *Proteobacteria*; *Zetaproteobacteria* | 1864480 | 55 | 47.58 | 58094 | 95.98 | 0 | Painkalac Creek, Australia | This study |
| PCRbin3 | PDZZ00000000 | *Proteobacteria*; *Lambdaproteobacteria* | 5059283 | 530 | 41.83 | 14906 | 80.25 | 0.31 | Punkally Creek, Australia | This study |
| WMHbin1 | PDZY00000000 | *Proteobacteria*; *Etaproteobacteria* | 4380192 | 242 | 54.3 | 24882 | 96.55 | 1.72 | Lake Weiming, China | This study |
| WMHbin3 | PDZX00000000 | *Proteobacteria*; *Etaproteobacteria* | 4601373 | 157 | 61.61 | 51212 | 98.28 | 1.72 | Lake Weiming, China | This study |
| WMHbin7 | PDZW00000000 | *Proteobacteria*; *Alphaproteobacteria* | 2984788 | 73 | 59.84 | 91589 | 94.83 | 0 | Lake Weiming, China | This study |
| WMHbinv6 | PDZV00000000 | *Proteobacteria*; *Etaproteobacteria* | 3839927 | 80 | 55.32 | 85378 | 92.16 | 0 | Lake Weiming, China | This study |
| YD0425bin50 | PDZT00000000 | *Proteobacteria*; *Deltaproteobacteira* | 4970388 | 589 | 36.91 | 11398 | 75.08 | 0 | Yuandadu Park, China | This study |
| YD0425bin51 | PDZS00000000 | *Proteobacteria*; *Deltaproteobacteira* | 5221616 | 134 | 32.23 | 82896 | 96.55 | 0 | Yuandadu Park, China | This study |
| YD0425bin7 | PDZU00000000 | *Proteobacteria*; *Etaproteobacteria* | 3583771 | 177 | 55.67 | 34600 | 94.83 | 0 | Yuandadu Park, China | This study |
| Omnitrophica_WOR_2_bacterium_GWA2_45_18 | MHFX00000000 | *Omnitrophica* | 2335743 | 24 | 46.36 | 133212 | 85.34 | 1.72 | Rifle well CD01, USA | Anantharaman *et al*. (2016) |
| Omnitrophica_WOR_2_bacterium_GWC2_45_7 | MHGD00000000 | *Omnitrophica* | 1340072 | 98 | 45.06 | 15451 | 67.48 | 1.72 | Rifle well CD01, USA | Anantharaman *et al*. (2016) |

^*^Genome completeness and contamination were estimated using CheckM (Parks *et al*., 2015) with the bacterial root marker set (104 markers).
