# Supplementary figures and images for: Genomic expansion of magnetotactic bacteria reveals an early common origin of magnetotaxis with lineage-specific evolution
Source: ISME J. 2018 Mar 26;12(6):1508–19. doi: 10.1038/s41396-018-0098-9 (PMC5955933; doi:10.1038/s41396-018-0098-9)

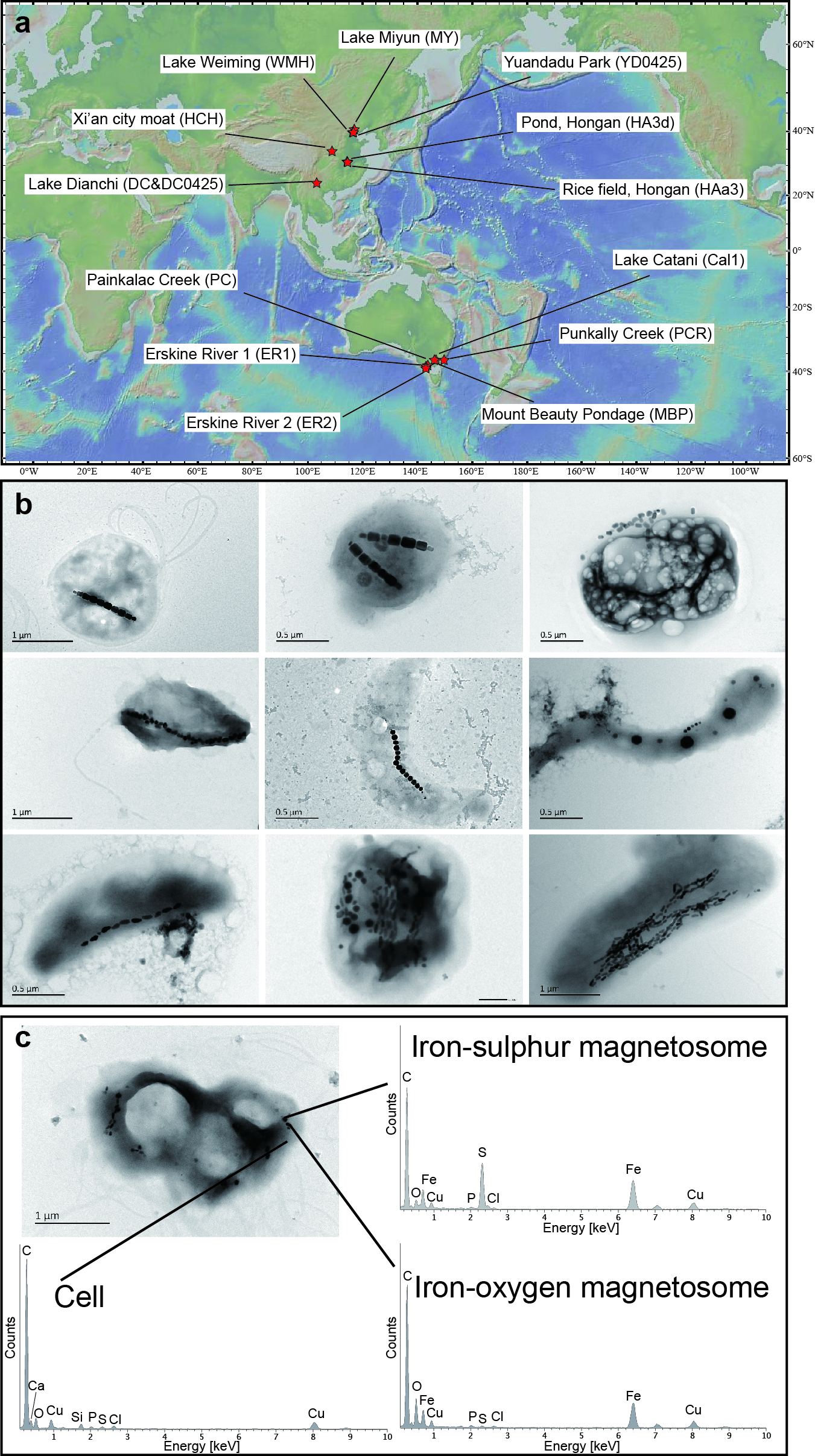

Supplement: Supplementary file 4 — Supplementary Figure 1(JPG 4220 kb) [file 41396_2018_98_MOESM4_ESM.jpg]

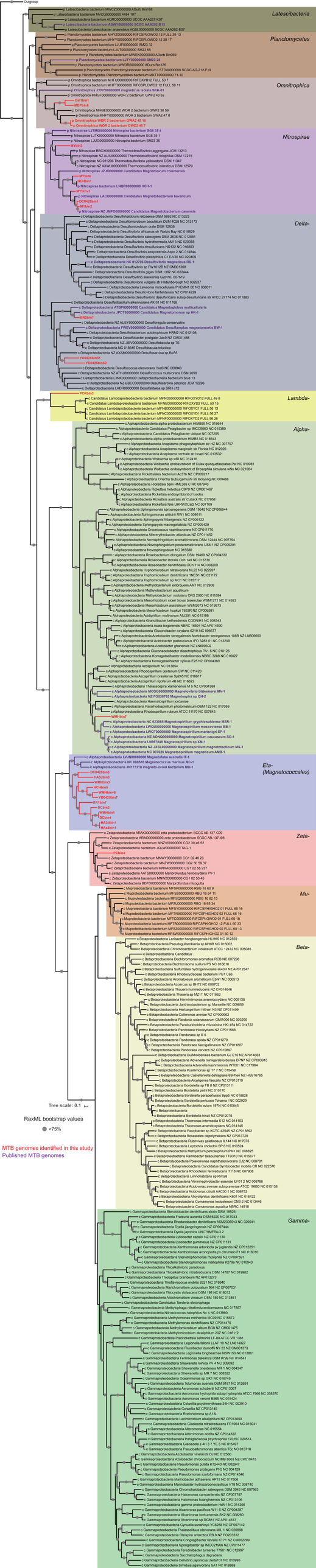

Supplement: Supplementary file 5 — Supplementary Figure 2(PDF 1650 kb) [file 41396_2018_98_MOESM5_ESM.pdf]

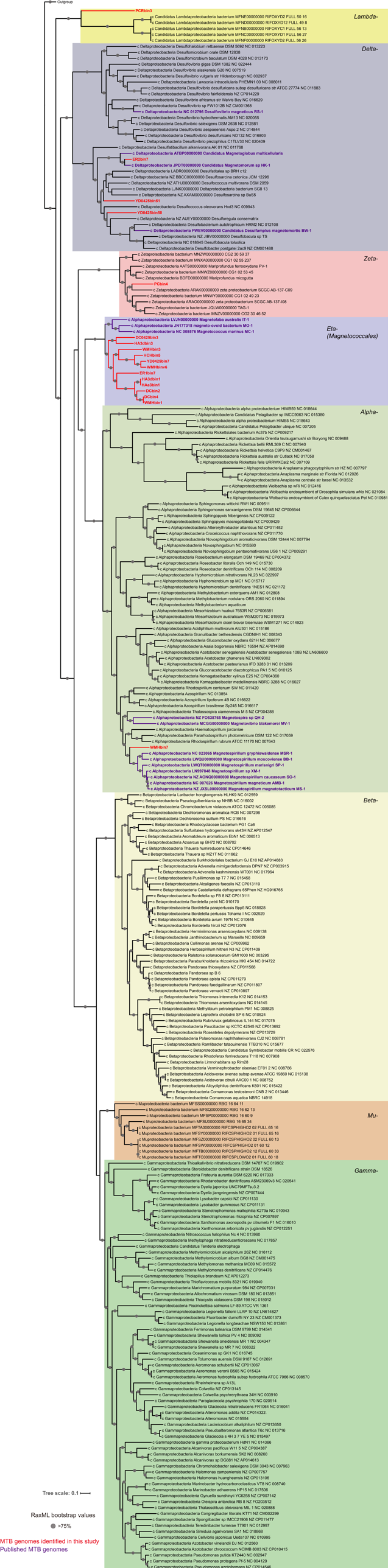

Supplement: Supplementary file 6 — Supplementary Figure 3(PDF 544 kb) [file 41396_2018_98_MOESM6_ESM.pdf]
